# Angiogenesis-facilitating and inflammation-modulating SIS-based patches coupled with functional peptides for abdominal wall repair

**DOI:** 10.1016/j.mtbio.2026.102795

**Published:** 2026-01-13

**Authors:** Zhenyu Zou, Yuchen Liu, Xueying Zhang, Jinxin Cao, Pengfei Wei, Yiqian Huang, Wei Jing, Bo Zhao, Minggang Wang

**Affiliations:** a Division of Hernia and Abdominal Wall Surgery, Department of General Surgery, Beijing Chao-Yang Hospital, Capital Medical University, 8 Gongren Tiyuchang Nanlu, Chaoyang District, Beijing, 100020, China; b Beijing Biosis Healing Biological Technology Co., Ltd., Daxing District, Beijing, 102600, China

**Keywords:** Abdominal wall repair, SIS, Peptide, Angiogenesis, Inflammation modulation

## Abstract

Abdominal wall defects caused by trauma, tumors, infections, abdominal surgery, and congenital factors can lead to functional impairments. The use of patches remains the most effective treatment approach. However, current repair materials still have limitations in regulating inflammation and promoting vascularization. Here, a small intestinal submucosa (SIS) extracellular patch was developed via conjugation with functional peptides PR1P and LL37 (*i.e.*, SIS-PR1P-LL37), to achieve angiogenesis and inflammation modulation for abdominal wall repair. In vitro experiments confirmed its superior pro-angiogenic potential when human umbilical vein endothelial cells (HUVECs) were treated with the patch, both tube formation (total tube length: 4.51 ± 0.53 mm, junction count: 28.00 ± 4.97) and scratch wound repair (repair area 3.26-fold that of the SIS group) outperformed the native SIS group (average tube length: ∼1.3 mm). After 7 days of culture, the VEGF expression was higher than that in the SIS group, and the expression levels of key angiogenic genes (VEGF, VEGFR-2, *etc.*) were 5.45–7.82-fold higher than those in the control group. Additionally, this peptide-conjugated SIS patch could enhance cell proliferation and angiogenic differentiation, effectively reduce the levels of inflammatory cytokines, and enrich the TLR and VEGF signaling pathways. The rat abdominal wall defect model further confirmed its superior capacity for tissue regeneration and angiogenesis, indicating it provides important theoretical and experimental support for the development of novel bioactive patches and holding promise for optimizing clinical strategies for abdominal wall repair.

## Graphical abstract

**Figure:**
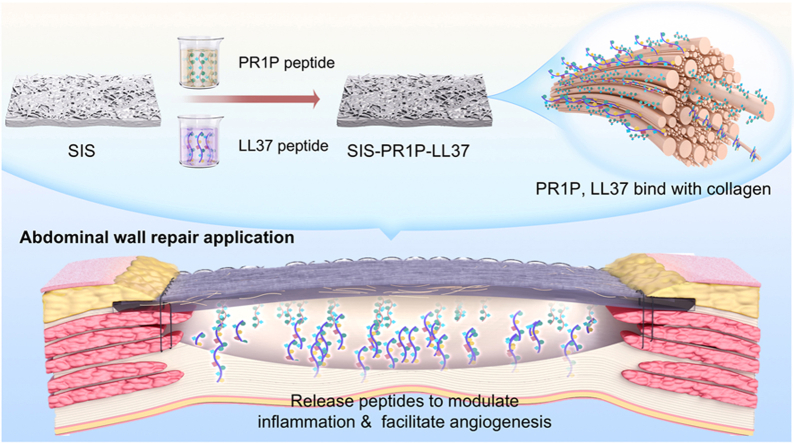

## 1 Introduction

Abdominal wall defects resulting from trauma, neoplasms, infections, abdominal surgeries, or congenital factors can lead to functional impairments that significantly compromise quality of life. Among these conditions, abdominal wall hernia is the most prevalent, with an estimated incidence of 14% [1]. The evolution of surgical techniques and biomaterials has established patch repair as the gold standard. However, achieving both anatomical reconstruction and functional restoration through patch-based repair remains a complex clinical challenge. Notably, while patch implantation significantly reduces recurrence rates, it concurrently increases the risk of patch-related complications [2]. Consequently, developing precise surgical strategies to minimize complications and optimize repair outcomes has emerged as a critical challenge in modern hernia surgery.

Clinically, repair of abdominal wall defects mainly relies on various patches, which can be classified into synthetic and biological materials [3]. Synthetic materials are widely used due to their mature manufacturing processes, low cost, and stable mechanical properties. However, they often exhibit poor tissue compatibility, triggering chronic inflammation and foreign body reactions that may cause various complications such as infection, adhesions, visceral erosion, and reduced abdominal wall compliance [[4], [5], [6], [7], [8], [9]]. In contrast, biological materials offer excellent biocompatibility and tissue integration, allowing them to blend more naturally with human tissue, promote regeneration and functional recovery, and reduce immune responses and postoperative complications [[10], [11], [12]]. Despite the significant biological advantages of biomaterial meshes, their clinical application still faces multiple challenges. First, the postoperative hernia recurrence rate is relatively high, and the long-term repair effect is not ideal [13,14]. Second, the mechanical strength and durability of the materials themselves are still inferior to those of synthetic materials, making it difficult to meet the repair needs of complex abdominal wall defects [[15], [16], [17]]. Third, the high preparation cost limits their popularization and application in clinical practice. More importantly, the current research and development as well as application of biomaterial meshes mostly focus on their physical support function, while the exploration of their key biological functions such as the regulation of local inflammatory microenvironment and the promotion of neovascularization remains insufficient [3,18,19]. A breakthrough in this field may provide an important direction for the innovation of abdominal wall defect repair technology.

In this study, we employed a small intestinal submucosa (SIS) decellularized extracellular matrix patch, whose surface is rich in active groups such as carboxyl and amino groups, providing a robust foundation for molecular modification. On this platform, we incorporated two complementary bioactive peptides, PR1P and LL37, based on their distinct yet synergistic roles in tissue repair. PR1P, a VEGF-binding peptide, was selected for its unique ability to stabilize and potentiate endogenous VEGF activity, thereby promoting sustained endothelial cell proliferation and migration [[20], [21], [22]]. This mechanism not only accelerates neovascularization but also ensures adequate oxygen and nutrient delivery to the regenerating tissue, which is essential for abdominal wall repair. LL37, an endogenous cationic antimicrobial peptide derived from human cathelicidin, was introduced for its multifaceted immunomodulatory functions. It can suppress excessive inflammation by modulating macrophage polarization toward an M2 phenotype, enhance epithelial regeneration, and simultaneously provide broad-spectrum antibacterial protection, reducing infection-related complications [23,24]. By limiting bacterial colonization at the implant site, LL37 may further stabilize the immunological microenvironment, indirectly supporting angiogenesis and constructive tissue remodeling. The combination of PR1P and LL37 was rationally designed to achieve a coordinated regulation of angiogenesis and immune balance, two interdependent processes that determine the quality of wound healing. While alternative angiogenic factors such as VEGF or FGF could promote vascularization, their short half-lives and potential for aberrant angiogenesis limit their practical application. Similarly, conventional antibiotics or anti-inflammatory agents lack the immunoregulatory precision and biocompatibility of LL37. Therefore, the dual-peptide modification strategy offers a biologically inspired and safer approach for precise intervention in vascular and immune responses during abdominal wall reconstruction.

First, the SIS patch was chemically treated to stabilize its surface groups. Then, a specific physicochemical method was used to immobilize PR1P and LL37 and enable their sustained release, ensuring continuous bioactivity throughout the repair process and overcoming the limitations of existing biological materials. Compared with biological materials loaded with bioactive cytokines or metallic ions [25,26], the advantages of using the SIS-PR1P-LL37 patch include the prolonged release of peptides as well as the avoidance of metallic accumulations to produce more compatible outcomes. The peptide-modified SIS patch developed in this study shows promising translational potential for clinical abdominal wall hernia repair. Clinically, the main challenges in achieving durable and complication-free hernia reconstruction arise from the initial ischemic and inflammatory microenvironment at the defect site, which compromises tissue integration and leads to complications such as infection, fibrosis, or recurrence. Our dual-functional modification strategy, integrating PR1P and LL37, directly targets these obstacles by synchronizing angiogenesis and immune regulation. Specifically, PR1P stabilizes endogenous VEGF and promotes sustained neovascularization, ensuring adequate oxygen and nutrient supply to the regenerating tissue. Meanwhile, LL37 exerts potent immunomodulatory effects by attenuating excessive inflammation and guiding macrophage polarization toward a reparative M2 phenotype, while also providing intrinsic antibacterial protection.

To validate this design, we conducted a series of experiments. Scanning electron microscopy (SEM) and confocal laser scanning microscopy (CLSM) confirmed that both PR1P and LL37 peptides were successfully and uniformly anchored to the surface of the SIS patch. In vitro angiogenesis experiments demonstrated that the modified patch significantly promoted endothelial cell proliferation, migration, and tubular structure formation, thereby confirming its pro-angiogenic effect. In an LPS-induced cellular inflammation model, the modified patch effectively regulated inflammatory cytokine levels, exhibiting strong immunomodulatory functions. RNA sequencing analysis further revealed that, in cells treated with the SIS-PR1P-LL37 patch, the toll-like receptor (TLR) and vascular endothelial growth factor (VEGF) signaling pathways were significantly enriched, providing molecular evidence for the observed effects. Finally, in vivo validation using a rat model of abdominal wall defect showed that the modified patch significantly accelerated wound healing, reduced inflammatory responses, and enhanced the formation of new vascular networks, demonstrating its potential for clinical application in abdominal wall repair.

## 2 Materials and methods

### 2.1 Materials

The SIS patches were sourced from Beijing Biosis Healing Biological Co., Ltd (Beijing, China). PR1P, LL37, and chimeric peptides were synthesized to 95% purity by Fmoc solid-phase peptide synthesis method (Jill Biochemical Shanghai Co., Ltd., China). Other chemicals, unless otherwise specified, were purchased from Beijing Chemical Reagent Co., Ltd. (China).

### 2.2 Preparation of SIS-PR1P-LL37 bio-patches

The SIS patches were cut into circular shapes to fit different well plate sizes. PR1P and LL37 were each prepared as 100 × 10^6^ M solutions. The SIS patch was immersed in the PR1P and LL37 solutions containing collagen linker peptides (TKKTLRT and KELNLVY, which bonded with collagen type Ⅰ and collagen type Ⅲ) for 2 h, followed by lyophilization to obtain the SIS-PR1P-LL37 patch, as previously reported [27]. The SIS-PR1P patch was immersed in the PR1P solutions containing collagen linker peptides for 2 h, followed by lyophilization to obtain the SIS-PR1P patch for further use. For fluorescent labelling and observation, the peptides PR1P-TKKTLRT and LL37-TKKTLRT were labeled with Rhodamine B, while the peptides PR1P-KELNLVY and LL37-KELNLVY were labeled with fluorescein isothiocyanate (FITC). TKKTLRT served as a specific binding peptide for collagen type I, and KELNLVY for collagen type Ⅲ, ensuring targeted attachment to the respective collagen components within the SIS patches.

### 2.3 Characterizations

After freeze-drying the SIS, SIS-PR1P, and SIS-PR1P-LL37 patches, they were sputter-coated with gold for 100 s and then examined under a scanning electron microscope (SEM, S4800, Hitachi) to analyze their surface morphology. For water contact angle measurements, the patches were fixed onto glass slides, and a 10 μL water droplet was deposited on the surface using an optical contact angle meter. Once the droplet stabilized, the contact angle was recorded. The peptide-releasing assay was conducted with a regular size (2 cm × 2 cm) of SIS-PR1P-LL37 patch immersed in 5 mL of deionized water. At time intervals (7, 14, 21, 28, and 35 days), the supernatants were retrieved, and the peptide release concentration was calculated by comparing them with a pre-established standard curve. The patches were shaped into regular sizes (3 cm × 1 cm) and mounted on a universal testing machine at a tensile speed of 5 mm/min for mechanical strength measurement at room temperature.

### 2.4 Cytocompatibility study

According to the ISO10993 standard, extract solutions were prepared at a sample-to-volume ratio of 6 cm^2^/mL. For cytocompatibility assessment, 2,000 cells per well were seeded in a 96-well plate, including L929 fibroblasts, human umbilical vein endothelial cells (HUVECs), and RAW264.7 macrophages. These cells were cultured on SIS, SIS-PR1P, and SIS-PR1P-LL37 scaffolds, and their proliferation activity was evaluated using the CCK-8 assay on days 1, 3, and 5.

For collagen production analysis, 5,000 L929 fibroblasts were seeded per well in a 24-well plate and cultured on SIS, SIS-PR1P, and SIS-PR1P-LL37 patches for 5 days. After fixation with 4% paraformaldehyde, the cells were incubated with primary antibodies against collagen type I and collagen type III, followed by secondary antibodies conjugated with FITC and Cy3, respectively. DAPI was used for nuclear staining, and collagen formation was visualized under CLSM. Cells cultured directly on tissue culture plates were used as the control group.

### 2.5 Angiogenic differentiation study

Following ISO10993 standards, SIS-based conditioned medium was prepared to culture HUVECs at a density of 5,000 cells per well. To assess tubular network formation, HUVECs were seeded onto Matrigel (Corning, USA), and cultured with the conditioned medium for 12 h. For the wound healing assay, when the cells reached ∼80% confluence, a scratch (∼200 μm) was established using a pipette tip. The cells were then cultured with the SIS-conditioned medium, and migration distance was measured after 12 h using an optical microscope.

For immunofluorescent staining assays, the HUVECs at a density of 5,000 cells per well were seeded on the patches. After seven days of culture, the cells were fixed and subjected to immunofluorescent staining using a VEGF primary antibody, FITC-labeled F-actin and DAPI. Images were captured under a CLSM. Total RNA was extracted using Trizol reagent (Invitrogen, USA) for reverse transcription polymerase chain reaction (RT-PCR) analysis.

For transcriptomic analysis, RNA-sequencing was performed on treated HUVECs. Cell lysates were extracted using Triton X-100, and sequencing was conducted on the Illumina X Ten platform. Quality control, raw read filtering, and genome mapping were performed to ensure data reliability. Gene expression levels were then quantified, and differential gene expression analysis was conducted to identify key genes. KEGG pathway enrichment analysis was carried out to determine the relevant signaling pathways.

### 2.6 Macrophage polarization study

RAW264.7 macrophages (10,000 cells per well) were pre-stimulated with LPS (1 μg mL^−1^) for 24 h before being cultured on different patches for three days. Immunofluorescent staining was conducted using antibodies against iNOS (ab178945, Abcam) and CD206 (PA5-101657, ThermoFisher). TNF-α and IL-10 levels were quantified using ELISA kits. Total RNA was extracted for RT-PCR analysis of pro-inflammatory and anti-inflammatory genes, including iNOS, TNF-α, IL-1β, IL-10, Arg-1 and CD206. Following LPS stimulation and subsequent culture on the patches, RNA-sequencing was performed on the treated macrophages. Cell lysates were extracted using Triton X-100, and sequencing was performed using the Illumina X Ten platform. Quality control, raw read filtering, genome mapping, differential expression analysis, and KEGG pathway enrichment analysis were carried out to investigate relevant pathways.

### 2.7 Animal study

All the animal experiments complied with the guidelines of the Tianjin Medical Experimental Animal Care, and animal protocols were approved by the Institutional Animal Care and Use Committee of Yi Shengyuan Gene Technology (Tianjin) Co., Ltd. Sprague-Dawley (SD) rats were anesthetized, and their abdominal area was disinfected with povidone-iodine. A 3 cm paramedian incision was made to avoid postoperative organ prolapse. The skin and subcutaneous tissue were separated, and a 2 cm × 1 cm partial-thickness abdominal wall defect was created, and the peritoneum was preserved. The experimental groups were as follows: 1) blank control (no implant), 2) SIS, 3) SIS-PR1P, 4) SIS-PR1P-LL37. The patches were secured with non-absorbable interrupted sutures, extending 0.5 cm beyond the defect margins. The incision was then closed. The abdominal wall tissue, along with the implanted patch, was excised and fixed in 4% paraformaldehyde for 48 h. After fixation, tissues were subjected to H&E staining and Masson's trichrome staining for histological analysis. Immunofluorescent staining of TNF-α (ab220210, Abcam), CD163 (ab182422, Abcam), CD31 (ab182981, Abcam), α-SMA (ab124964, Abcam), collagen type I (ab254113, Abcam), and collagen type Ⅲ (ab6310, Abcam) was also conducted on the harvested tissues. At week 12, major organs including heart, liver, spleen, lung and kidney were collected for systemic biocompatibility study using H&E staining.

### 2.8 Statistical analysis

All quantitative data were represented as mean ± standard deviation (SD). Statistical analysis was performed using SPSS software, employing one-way analysis of variance (ANOVA). Differences were considered statistically significant at ∗*P* ≤ 0.05, highly significant at ∗∗*P* ≤ 0.01, and very highly significant at ∗∗∗*P* ≤ 0.001.

## 3 Results and discussion

### 3.1 Preparation and characterization of the SIS-PR1P-LL37 patch

**Fig. 1:**
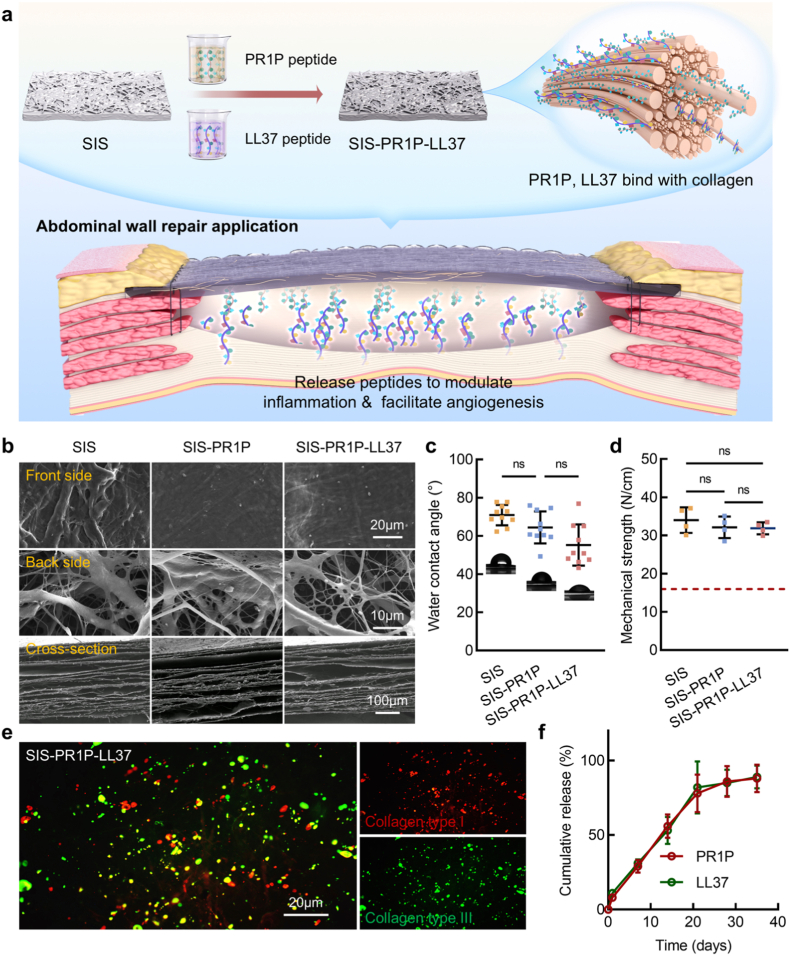
Characterization of the peptide-coupled SIS-based patch for abdominal wall repair. (a) Schematic illustration of the fabrication process for the SIS-PR1P-LL37 patch designed for abdominal wall repair. The PR1P and LL37 peptides were sequentially anchored on the SIS matrix collagen. Upon implantation, the SIS-PR1P-LL37 facilitated tissue regeneration by releasing PR1P and LL37 peptides to enhance angiogenesis and modulate inflammation. (b) SEM images for the microstructures of SIS, SIS-PR1P, and SIS-PR1P-LL37 patches. (c) Water contact angle measurements for the SIS, SIS-PR1P, and SIS-PR1P-LL37 patches (n = 10). (d) Mechanical strength of the SIS, SIS-PR1P, and SIS-PR1P-LL37 patches (n = 4). (e) CLSM fluorescent images staining with collagen type Ⅰ and collagen type Ⅲ to visualize the binding of PR1P and LL37 peptides. (f) Cumulative release of PR1P and LL37 peptides from the SIS-PR1P-LL37 patch.

Native SIS mainly serves as a permissive ECM scaffold, while its biological instructiveness is significantly enhanced through peptide functionalization. The SIS-PR1P-LL37 patch was fabricated by immersing the SIS patch in aqueous solutions of PR1P and LL37 peptides, followed by lyophilization. The incorporation of PR1P and LL37 peptides onto the collagen fibers of SIS aimed to enhance its pro-angiogenic and immunomodulatory functions. Upon implantation at the abdominal wall defect site, SIS served as a structural scaffold for peptide delivery, allowing the sustained release of PR1P and LL37 to promote vascularization and modulate the local inflammatory response (Fig. 1a). The morphology of the lyophilized SIS-PR1P-LL37 patch was shown in Fig. 1b. The patch maintained its native structural composition and multi-layered structure, as characterized by the presence of thick collagen type I and finer collagen type III fibers, which closely resembled the original SIS structure [28]. This observation confirmed that the peptide-immersion process did not significantly alter the microstructure of SIS. Additionally, the peptide modification enhanced the patch’s hydrophilicity, as indicated by a progressive decrease in water contact angles: SIS (71.82 ± 5.90°), SIS-PR1P (62.99 ± 9.27°), and SIS-PR1P-LL37 (54.14 ± 10.89°), though showing no statistical difference between SIS and SIS-PR1P (Fig. 1c). The mechanical strength values of the SIS-based patches were 34.02 ± 3.35 N/cm (SIS), 32.13 ± 2.83 N/cm (SIS-PR1P), 31.88 ± 1.58 N/cm (SIS-PR1P-LL37), respectively, and all the patches met the standard line for abdominal wall application [29], without showing statistical significance associated with peptide modification (Fig. 1d). Furthermore, fluorescence staining revealed the integration of PR1P and LL37 peptides with collagen type I and collagen type III fibers within the SIS (Fig. 1e), as well as the constant release of PR1P and LL37 peptides till 28 days (Fig. 1f), confirming the stable preparation of the SIS-PR1P-LL37 patch.

### 3.2 In vitro evaluation of the SIS-PR1P-LL37 patch

**Fig. 2:**
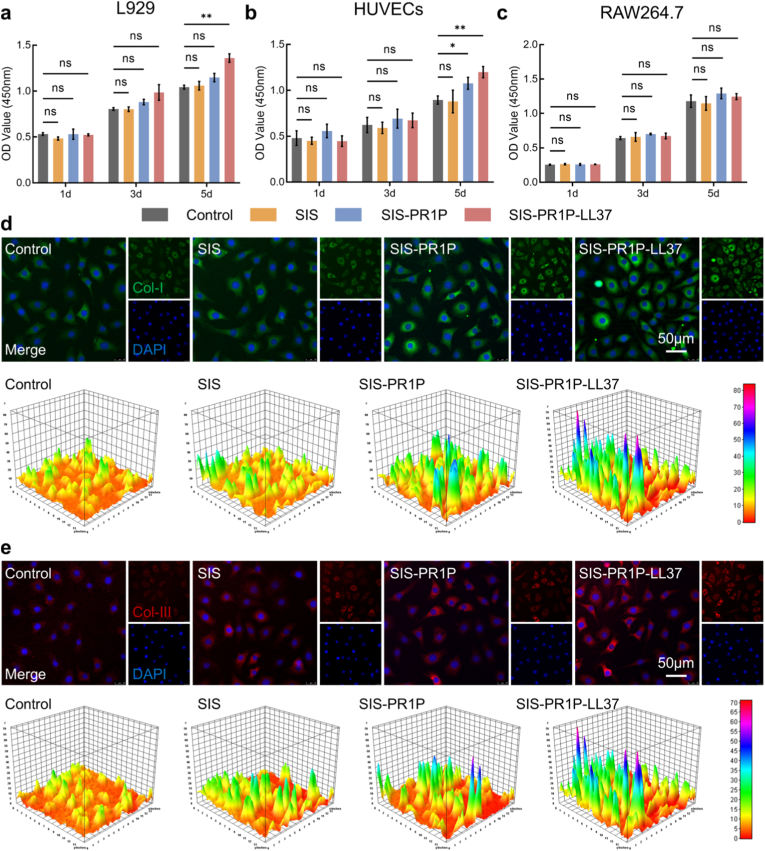
Cytocompatibility and collagen deposition of the SIS-PR1P-LL37 patch. (a–c) L929 fibroblasts, HUVECs, and RAW264.7 macrophages were seeded on the SIS-PR1P-LL37 patch and cultured for 5 days to assess cytocompatibility. Immunofluorescent staining of (d) collagen type Ⅰ and (e) collagen type Ⅲ in L929 fibroblasts after five days of culture, along with corresponding 3D reconstruction images. Statistical significance and *P* values were determined by ANOVA comparison test. The data were presented as mean ± SD (n = 4), ∗*P* ≤ 0.05, ∗∗*P* ≤ 0.01.

To assess the biocompatibility of the SIS patches, L929 fibroblasts, HUVECs, and RAW264.7 macrophages were seeded onto the SIS, SIS-PR1P, and SIS-PR1P-LL37 patches, with cells cultured on the tissue culture plates as the control. Cell viability was analyzed using the CCK-8 assay over a 5-day culture period. The results demonstrated that the SIS-PR1P-LL37 patch promoted cell proliferation, exhibiting no significant cytotoxicity compared to the control (Fig. 2(a–c)).

Furthermore, to evaluate the patch’s effect on extracellular matrix remodeling, immunofluorescent staining of collagen type I and collagen type III was performed on the L929 fibroblasts after 5 days of culture. The results revealed a marked increase in collagen secretion in cells cultured on SIS-PR1P-LL37 (Fig. 2(d, e)), indicating its potential to promote tissue repair. These findings suggested that the SIS-PR1P-LL37 patch provided a supportive microenvironment for cellular growth and extracellular matrix deposition.

### 3.3 Evaluation of the angiogenic potential of the SIS-PR1P-LL37 patch

**Fig. 3:**
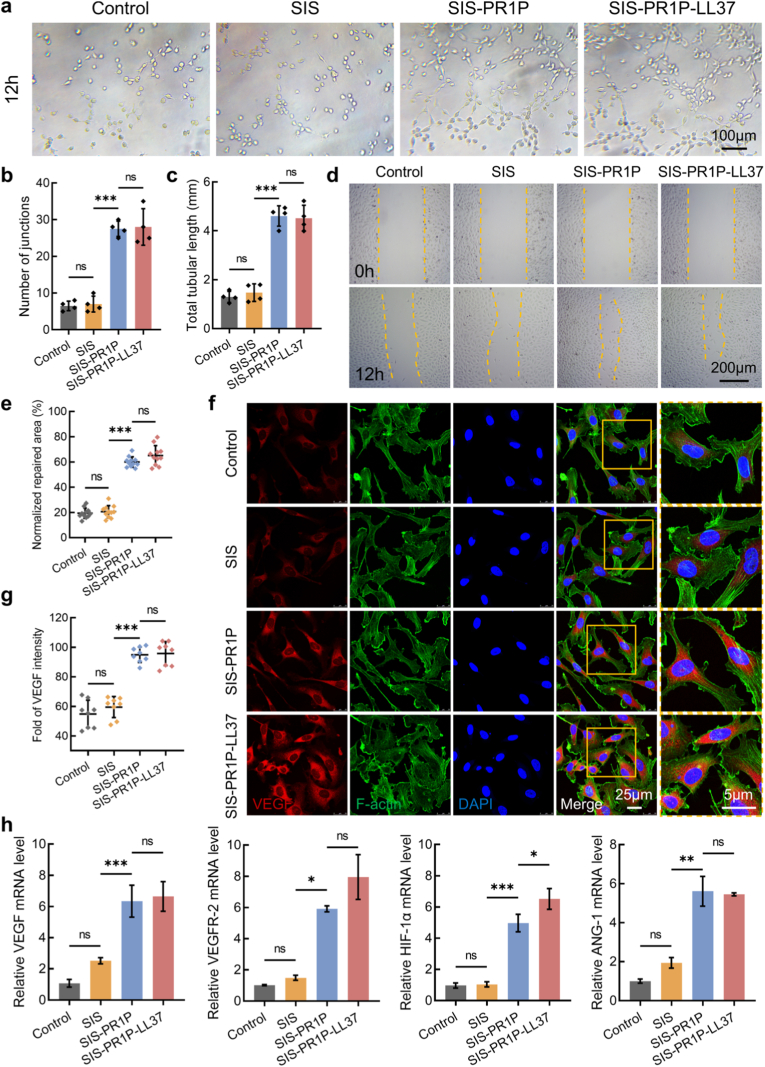
Pro-angiogenic potential of the SIS-PR1P-LL37 patch. (a) Microtubular formation assay using the leaching extracts to co-culture with HUVECs for 12 h, and corresponding estimate of (b) number of junctions and (c) total tubular length. (d) Scratch wound healing assays using HUVECs and the leaching extracts to monitor the cell migration for 12 h, and (e) semi-quantitative analysis of the repaired area. (f) Immunofluorescent staining of VEGF (red), F-actin (green) and DAPI (blue) in HUVECs cultured on the patches for seven days, and (g) their fluorescent intensities. (h) RT-PCR analysis for the angiogenesis- and inflammation-related genes including VEGF, VEGFR-2, HIF-α and ANG-1, for those HUVECs being cultured for seven days on the patches. Statistical significance and *P* values were determined by ANOVA comparison test. The data were presented as mean ± SD (n = 4), ∗*P* ≤ 0.05, ∗∗*P* ≤ 0.01, ∗∗∗*P* ≤ 0.001. (For interpretation of the references to colour in this figure legend, the reader is referred to the Web version of this article.)

To assess the pro-angiogenic potential of the SIS-PR1P-LL37, we firstly cultured HUVECs with the extract solutions of different patches. In the tube formation assay, HUVECs were pre-seeded on Matrigel and incubated with different extract solutions for 12 h. The SIS-PR1P and SIS-PR1P-LL37 groups exhibited significantly higher junction numbers (27.50 ± 2.08 and 28.00 ± 4.97, respectively) and total tube lengths (4.61 ± 0.42 mm and 4.51 ± 0.53 mm, respectively) compared to the control and SIS groups, which displayed an average tube length of approximately 1.3 mm (Fig. 3(a–c)). Similarly, in the HUVEC scratch assay, the extract solutions from the SIS-PR1P and SIS-PR1P-LL37 significantly enhanced cell migration, with the repaired area being approximately 3.00-fold and 3.26-fold higher than that of the control and SIS (Fig. 3(d, e)). After seven days of incubation on the patches, VEGF immunofluorescent staining demonstrated that the SIS-PR1P and SIS-PR1P-LL37 induced significantly higher VEGF expression compared to the control and SIS (Fig. 3(f, g)). Furthermore, RT-PCR analysis revealed that the SIS-PR1P-LL37 significantly up-regulated key angiogenic genes, with VEGF, VEGFR-2, HIF-1α and ANG-1 expression levels being 6.21-, 7.82-, 6.73-, and 5.45-fold higher than those in the control group (Fig. 3h), indicating that the SIS-PR1P-LL37 effectively promoted angiogenic differentiation and accelerated tissue vascularization.

### 3.4 Inflammation modulation by the SIS-PR1P-LL37 patch

**Fig. 4:**
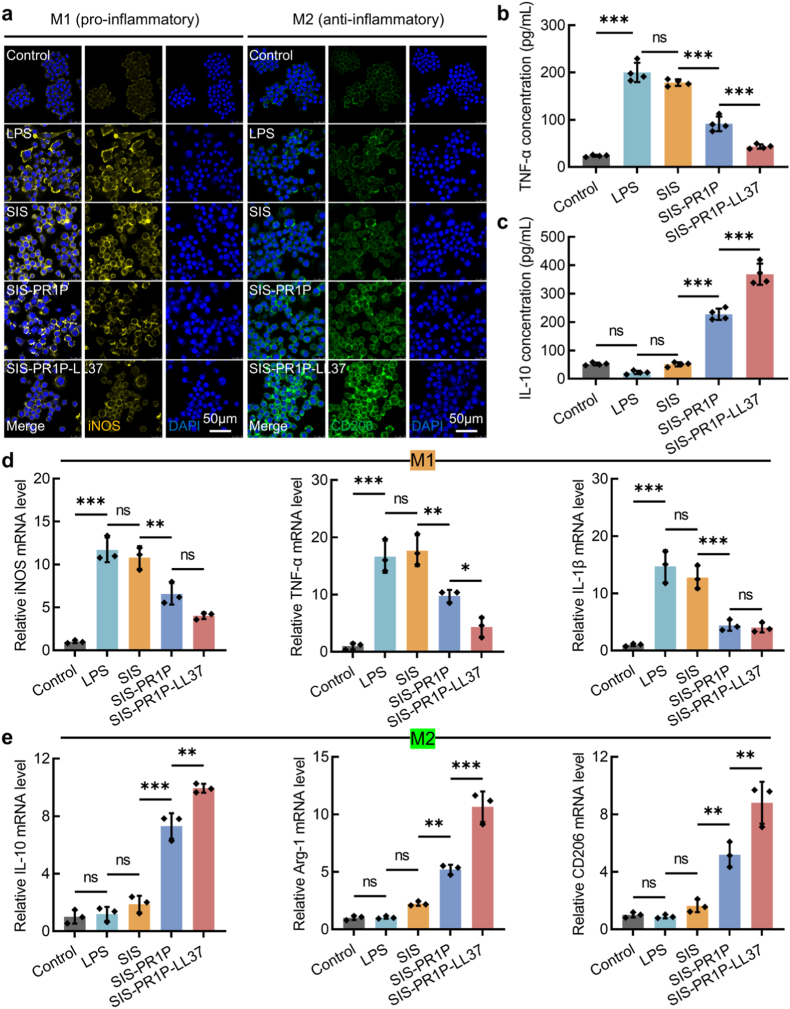
Inflammation modulation by the SIS-PR1P-LL37 patch. (a) Immunofluorescent staining of iNOS (yellow, M1) and CD206 (green, M2) in the RAW264.7 macrophages pre-treated with LPS for 24 h and cultured on the SIS-PR1P-LL37 patch for 3 days. ELISA quantification of (b) TNF-α (pro-inflammatory, M1) and (c) IL-10 (anti-inflammatory, M2) cytokines. The data were presented as mean ± SD (n = 4). RT-PCR analysis of inflammation-related genes including (d) M1 markers: iNOS, TNF-α, IL-1β and (e) M2 markers: IL-10, Arg-1, CD206 for 3 days. The data were presented as mean ± SD (n = 3). Statistical significance and *P* values were determined by ANOVA comparison test. ∗*P* ≤ 0.05, ∗∗*P* ≤ 0.01, ∗∗∗*P* ≤ 0.001. (For interpretation of the references to colour in this figure legend, the reader is referred to the Web version of this article.)

To evaluate the immunomodulatory effects of the SIS-PR1P-LL37 patch, we established an in vitro inflammation model using RAW264.7 macrophages stimulated with LPS for 24 h [30]. Following LPS stimulation, a strong inflammatory response was observed, as characterized by a significant increase in iNOS fluorescent intensity. However, treatment with SIS-PR1P-LL37 significantly reduced iNOS expression while markedly enhancing the fluorescent intensity of the anti-inflammatory marker CD206 (Fig. 4a). ELISA and RT-PCR were further used to analyze the inflammatory response. ELISA results showed a substantial increase in TNF-α levels following LPS stimulation, whereas IL-10 remained unchanged. However, the SIS-PR1P-LL37 group significantly reduced TNF-α levels (200.20 ± 20.37 pg/mL post-LPS stimulation compared to 43.32 ± 4.77 pg/mL) and markedly increased IL-10 levels (22.05 ± 5.88 pg/mL post-LPS stimulation compared to 368.34 ± 37.46 pg/mL) (Fig. 4(b, c)). RT-PCR analysis further confirmed that the expression of pro-inflammatory markers, including iNOS, TNF-α, and IL-1β, was significantly down-regulated in the SIS-PR1P-LL37 group compared to the LPS-stimulated group. Conversely, anti-inflammatory markers such as IL-10, Arg-1, and CD206 were markedly up-regulated in the SIS-PR1P-LL37 group (Fig. 4(d, e)), demonstrating its potent inflammation-modulating effects. Such dual modulation was more consistent with clinical demands for sustained vascular support, reduced fibrosis, and minimized infection risk in hernia repair. This advantage, while inspired by recent progress in macrophage-polarizing or ECM-remodeling materials [31], underscored the translational promise of our SIS-PR1P-LL37 system as a safe and tunable platform for complex abdominal wall reconstruction.

### 3.5 Mechanistic insights into the SIS-PR1P-LL37 regulation of cell behavior

**Fig. 5:**
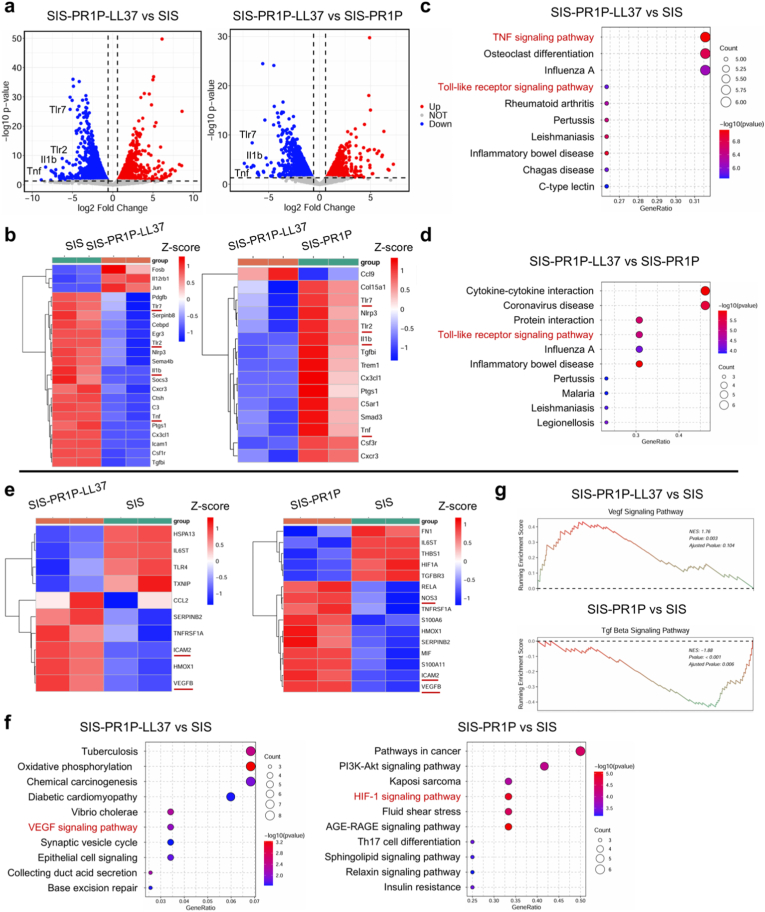
Molecular mechanism underlying the SIS-PR1P-LL37 patch function via RNA-sequencing. (a) Volcano plots depicting the differential genes of RAW264.7 between SIS-PR1P-LL37 vs SIS, and SIS-PR1P-LL37 vs SIS-PR1P. (b) Heatmap of gene clusters between SIS-PR1P-LL37 vs SIS, and SIS-PR1P-LL37 vs SIS-PR1P. KEGG pathway enrichment analysis comparing (c) SIS-PR1P-LL37 vs SIS, and (d) SIS-PR1P-LL37 vs SIS-PR1P. (e) Heatmaps of gene clusters in HUVECs between SIS-PR1P-LL37 vs SIS, and SIS-PR1P vs SIS-PR1P. (f) KEGG pathway enrichment analysis of the SIS-PR1P-LL37 vs SIS, and SIS-PR1P vs SIS. (g) GSEA enrichment curves highlighting key signaling pathways involved in angiogenesis and inflammation modulation.

To elucidate the molecular mechanisms underlying the effects of the SIS-PR1P-LL37 on RAW264.7 macrophages and HUVECs, we performed RNA sequencing analysis. Differential gene expression analysis revealed significant differences between SIS-PR1P-LL37, SIS-PR1P, and SIS (Fig. 5(a, b)). Notably, the SIS-PR1P-LL37 led to the down-regulation of key inflammatory genes, such as TLR2, TLR7, and TNF. KEGG enrichment analysis indicated that SIS-PR1P-LL37 modulated inflammatory responses through the TNF signaling pathway and the TLR signaling pathway (Fig. 5(c, d)).

In terms of angiogenic regulation, the SIS-PR1P-LL37 significantly altered the expression of vascularization-related genes, including VEGFB, NOS3, and ICAM2. KEGG pathway analysis further demonstrated that these genes were enriched in the VEGF signaling pathway (Fig. 5(e–g)), providing mechanistic insights into the pro-angiogenic effects of SIS-PR1P-LL37. These findings highlighted the molecular targets by which the SIS-PR1P-LL37 promoted angiogenesis and modulated inflammation, offering a promising strategy for tissue repair and regeneration.

### 3.6 Evaluation of the SIS-PR1P-LL37 for abdominal wall defect repair

**Fig. 6:**
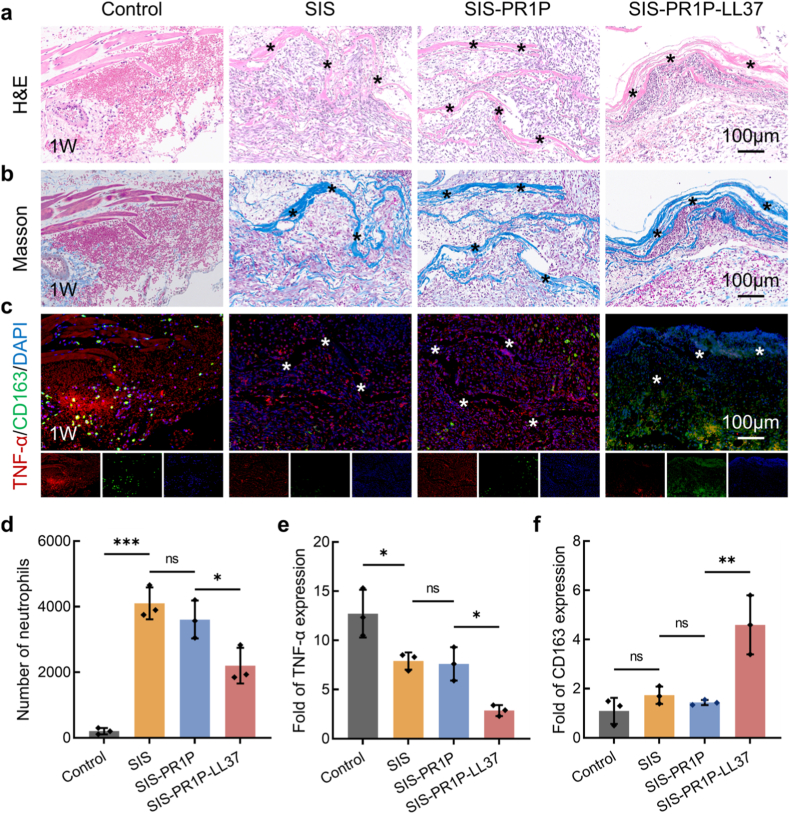
Abdominal wall repair after 1 week of implantation. (a) H&E staining to assess the early stage of implantation and histocompatibility. (b) Masson's trichrome staining at the implantation site to evaluate collagen deposition. (c) Immunofluorescent staining of TNF-α (red), CD163 (green) and DAPI (blue) at the implantation site. (d) Numbers of neutrophils based on panel (a). Semi-quantitative analysis of (e) TNF-α and (f) CD163 fluorescent intensity based on panel (c). The asterisk indicates the implanted patch. Statistical significance and *P* values were determined by ANOVA comparison test. The data were presented as mean ± SD (n = 3), ∗*P* ≤ 0.05, ∗∗*P* ≤ 0.01, ∗∗∗*P* ≤ 0.001. (For interpretation of the references to colour in this figure legend, the reader is referred to the Web version of this article.)

To evaluate the implantation efficacy and tissue regeneration potential of the SIS-PR1P-LL37, we established a rat abdominal wall defect model and implanted different patches. During the early implantation stage (week 1), the introduction of patches invoked significant neutrophil recruitment due to innate immune rejection. However, the SIS-PR1P-LL37 group exhibited significantly fewer neutrophils compared to the SIS and SIS-PR1P groups (Fig. 6(a, d)). Masson’s trichrome staining revealed initial collagen deposition around the SIS-PR1P-LL37, whereas the control and SIS groups displayed minimal newly formed collagen (Fig. 6b). At this stage, the expression of TNF-α remained high in the control, SIS, and SIS-PR1P groups, which could hinder wound healing. In contrast, the SIS-PR1P-LL37 showed a lower neutrophil count (0.54-fold to the SIS), reduced TNF-α expression (0.22-fold to the control), and significantly increased anti-inflammatory marker CD163 expression (4.18-fold to the control) (Fig. 6(c–e, f)), laying the foundation for subsequent tissue repair.

**Fig. 7:**
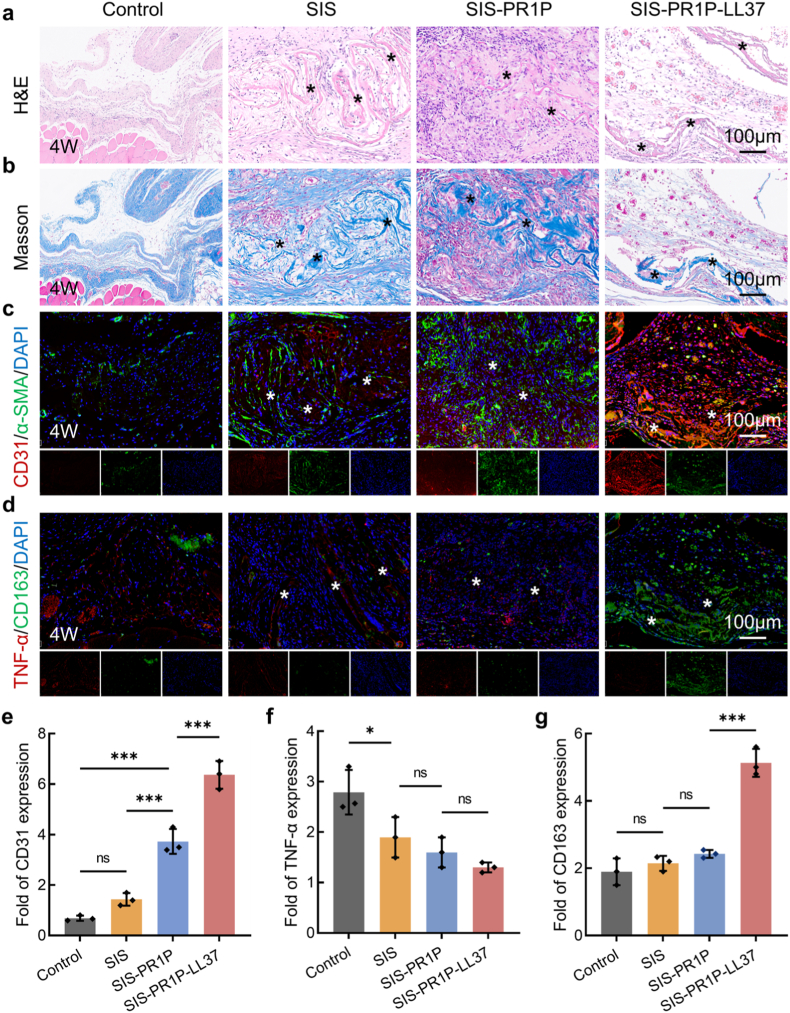
Abdominal wall repair after 4 weeks of implantation. (a) H&E staining to evaluate the histocompatibility and biodegradation of the implanted patch. (b) Masson's trichrome staining at the implantation site. (c) Immunofluorescent staining of CD31 (red), α-SMA (green), and DAPI (blue) at the implantation site. (d) Immunofluorescent staining of TNF-α (red), CD163 (green) and DAPI (blue) at the implantation site. Semi-quantitative analysis of (e) CD31, (f) TNF-α, and (g) CD163 fluorescent intensity based on panels (c, d). The asterisk indicates the implanted patch. Statistical significance and *P* values were determined by ANOVA comparison test. The data were presented as mean ± SD (n = 3), ∗*P* ≤ 0.05, ∗∗∗*P* ≤ 0.001. (For interpretation of the references to colour in this figure legend, the reader is referred to the Web version of this article.)

At week 4 post-implantation, partial degradation of the patch was observed, with its initial dense fibrous structure becoming looser. Notably, no excessive neutrophil aggregation was detected (Fig. 7a). Masson's trichrome staining revealed pronounced neovascularization in the SIS-PR1P and SIS-PR1P-LL37 groups (Fig. 7b). Immunofluorescent staining showed that the angiogenic marker CD31 expression in the SIS-PR1P group was 2.60-fold and 5.44-fold higher than that in the SIS and control groups, respectively. The SIS-PR1P-LL37 group exhibited even greater CD31 expression, which was 1.71-fold higher than that in the SIS-PR1P group and 9.27-fold higher than that in the control group, indicating a strong correlation between early inflammation modulation and enhanced angiogenesis. Similarly, the TNF-α and CD163 immunofluorescent staining confirmed that TNF-α expression remained low in the SIS-PR1P-LL37 group, while CD163 expression remained high (Fig. 7(c–g)).

**Fig. 8:**
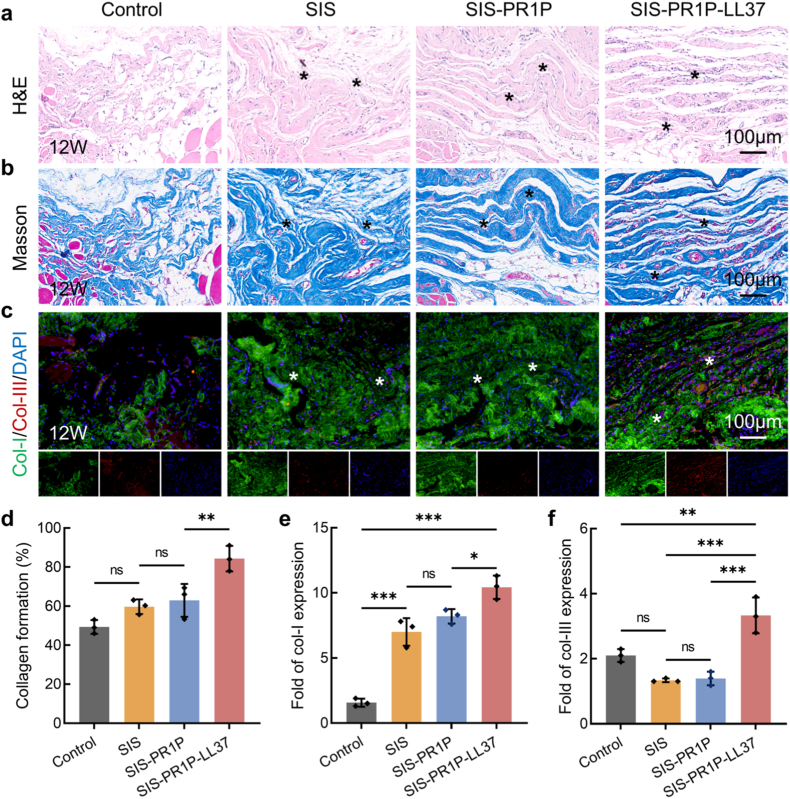
Abdominal wall repair after 12 weeks of implantation. (a) H&E staining to evaluate the histocompatibility and long-term biodegradation in vivo. (b) Masson's trichrome staining to assess collagen formation. (c) Immunofluorescent staining of collagen type Ⅰ (green), collagen type Ⅲ (red) and DAPI (blue) at the implantation site. Semi-quantitative analysis of (d) collagen formation, (e) collagen type Ⅰ, and (f) collagen type Ⅲ expressions based on panels (b, c). The asterisk indicates the implanted patch. Statistical significance and *P* values were determined by ANOVA comparison test. The data were presented as mean ± SD (n = 3), ∗*P* ≤ 0.05, ∗∗*P* ≤ 0.01, ∗∗∗*P* ≤ 0.001. (For interpretation of the references to colour in this figure legend, the reader is referred to the Web version of this article.)

By week 12, significant degradation of the SIS patch was observed, with only minimal residual material remaining (Fig. 8a). However, in the SIS-PR1P-LL37 group, collagen deposition was denser and more organized, with a total collagen content 1.71-fold and 1.41-fold higher than that of the control and SIS, respectively (Fig. 8(b–d)). Immunofluorescent staining for type I and type III collagen demonstrated that the SIS-PR1P-LL37 promoted higher collagen expression (6.66-fold and 1.59-fold higher than the control, respectively), confirming its superior efficacy in facilitating abdominal wall regeneration (Fig. 8(c–e)).

**Fig. 9:**
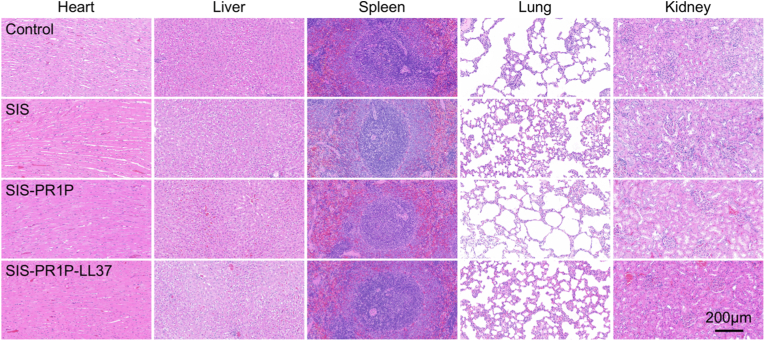
Biocompatibility study. (a) Tissue adhesion scoring standard. (b) Tissue adhesion score after the patches were implanted to the abdominal wall. (c) Histological staining of major organs including heart, liver, spleen, lung and kidney for the SIS-PR1P-LL37 patch implanted to the abdominal wall for 12 weeks.

Given the biodegradable nature of SIS, we performed histopathological examinations of major organs (heart, liver, spleen, lung, and kidney) after 12 weeks of implantation. The results indicated no adverse effect on these organs, with the tissue morphology from the SIS-PR1P-LL37 group closely resembling that of the control (Fig. 9), demonstrating its excellent biocompatibility.

Although the SIS-PR1P exhibited significant pro-angiogenic activity in the early stage, it failed to effectively induce collagen type III deposition and even performed worse than the control. This might be attributed to its rapid induction of angiogenesis without effectively modulating inflammation at the injury site, which remains an area for further investigation. Unraveling the precise mechanism behind this phenomenon would be a key focus of our next research.

## 4 Conclusions

In this study, we anchored the pro-angiogenic and inflammation-regulating peptides PR1P and LL37 onto the SIS patch through immersion in chimeric peptide solutions. In vitro cell experiments confirmed that the SIS-PR1P-LL37 exhibited excellent pro-angiogenic and inflammation-modulating effects. RNA sequencing further revealed that the SIS-PR1P-LL37 regulated these processes through TLR and VEGF signaling pathways. In a rat abdominal wall defect model, the SIS-PR1P-LL37 demonstrated superior collagen production and tissue regeneration capacity, with collagen deposition 1.71-fold higher than the control. These findings established the SIS-PR1P-LL37 patch as a promising novel biomaterial for abdominal wall repair.

## CRediT authorship contribution statement

**Zhenyu Zou:** Writing – original draft, Investigation, Data curation, Conceptualization. **Yuchen Liu:** Investigation, Conceptualization. **Xueying Zhang:** Investigation, Formal analysis. **Jinxin Cao:** Validation. **Pengfei Wei:** Validation. **Yiqian Huang:** Validation. **Wei Jing:** Validation. **Bo Zhao:** Writing – review & editing, Methodology. **Minggang Wang:** Writing – review & editing, Supervision, Project administration.

## Declaration of competing interest

The authors declare that they have no known competing financial interests or personal relationships that could have appeared to influence the work reported in this paper.

## Data Availability

Data will be made available on request.
